# Supplementary material for: Socio-economic inequalities in the breadth of internet use before and during the COVID-19 pandemic among older adults in England
Source: PLoS One. 2024 May 9;19(5):e0303061. doi: 10.1371/journal.pone.0303061 (PMC11081243; doi:10.1371/journal.pone.0303061)
Supplement: S1 Table — Note: ELSA, English Longitudinal Study of Ageing. Survey question at baseline: On average, how often do you use the internet or email? Survey question at follow-up: Since the coronavirus outbreak, on average, how often did you use the internet or email? (DOCX) [file pone.0303061.s002.docx]

| **Categories** | **Response options at baseline (wave nine of the main ELSA survey)** | **Response options at follow-up (wave one of the ELSA COVID-19 sub-study)** |
| --- | --- | --- |
| *High:*  *Moderate:*  *Low:*  *Never:* | - Every day, or almost every day - At least once a week (but not every day) - At least once a month (but not every week) - At least once every three months - Less than every three months - Never | - More than once a day - Every day, or almost every day - At least once a week (but not every day) - At least once a month (but not every week) - Less than monthly - Never |
