# Supplementary material for: Socio-economic inequalities in the breadth of internet use before and during the COVID-19 pandemic among older adults in England
Source: PLoS One. 2024 May 9;19(5):e0303061. doi: 10.1371/journal.pone.0303061 (PMC11081243; doi:10.1371/journal.pone.0303061)
Supplement: S2 Table — Note: ELSA, English Longitudinal Study of Ageing. Survey question at baseline and follow-up: For which of the following activities did you use the internet in the last 3 months? (DOCX) [file pone.0303061.s003.docx]

| **Categories** | **Response options at baseline (wave nine of the main ELSA survey)** | **Response options at follow-up (wave one of the ELSA COVID-19 sub-study)** |
| --- | --- | --- |
| *Emails* | - Sending/receiving emails | - Sending/receiving emails |
| *Calls* | - Telephoning over the internet/video calls (via webcam) over the internet | - Making video calls or voice calls (using applications such as Skype, WhatsApp, or FaceTime) |
| *Health* | - Finding information on health-related issues | - Finding information on health-related issues |
| *Entertainment* | - Streaming/downloading live or on demand TV/radio (BBC iPlayer, Netflix, Amazon Prime Video), music (iTunes, Spotify, Apple Music), or ebooks - Games | - Streaming TV/videos/radio (BBC iPlayer, Netflix, Amazon Prime, YouTube), listening to music (Spotify, Apple Music), playing online games, or reading ebooks |
| *News* | - News/newspaper/blog websites | - Reading news/newspaper/blog websites |
| *Market* | - Shopping/buying goods or services | - Shopping/buying goods or services |
| *Social networking* | - Using social networking sites (Facebook, Twitter, LinkedIn, Instagram) - Creating, uploading, or sharing content (YouTube, blogging or Flickr) | - Using social networking sites (Facebook, Twitter, LinkedIn, Instagram, blogging or Flickr) |
| *Internet transactions* | - Finances (banking, paying bills) - Using public services (e.g., obtaining benefits, paying taxes) | - Managing my finances (online banking, paying bills, paying taxes) |
