# Supplementary material for: Socio-economic inequalities in the breadth of internet use before and during the COVID-19 pandemic among older adults in England
Source: PLoS One. 2024 May 9;19(5):e0303061. doi: 10.1371/journal.pone.0303061 (PMC11081243; doi:10.1371/journal.pone.0303061)
Supplement: S3 Table — Note: n, number of participants. Bold denotes statistical significance (p<0.05). (DOCX) [file pone.0303061.s004.docx]

| **Online activities** | **Male participants**  ***n* (%)** | **Female participants**  ***n* (%)** | $\boldsymbol{\chi}$**^2^** | ***p* value** |
| --- | --- | --- | --- | --- |
| **Emails** |  |  | 7.27 | **0.007** |
| Yes | 1,624 (89.3) | 1,933 (86.5) |  |  |
| No | 195 (10.7) | 302 (13.5) |  |  |
| **Calls** |  |  | 0.30 | 0.585 |
| Yes | 556 (30.6) | 701 (31.4) |  |  |
| No | 1,263 (69.4) | 1,534 (68.6) |  |  |
| **Health** |  |  | 24.34 | **<0.001** |
| Yes | 1,505 (82.7) | 1,708 (76.4) |  |  |
| No | 314 (17.3) | 527 (23.6) |  |  |
| **Entertainment** |  |  | 0.00 | 0.977 |
| Yes | 975 (53.6) | 1,199 (53.6) |  |  |
| No | 844 (46.4) | 1,036 (46.4) |  |  |
| **News** |  |  | 52.01 | **<0.001** |
| Yes | 1,021 (56.1) | 1,000 (44.7) |  |  |
| No | 798 (43.9) | 1,235 (55.3) |  |  |
| **Market** |  |  | 29.32 | **<0.001** |
| Yes | 1,388 (76.3) | 1,534 (68.6) |  |  |
| No | 431 (23.7) | 701 (31.4) |  |  |
| **Social networking** |  |  | 44.78 | **<0.001** |
| Yes | 749 (41.2) | 1,156 (51.7) |  |  |
| No | 1,070 (58.8) | 1,079 (48.3) |  |  |
| **Internet transactions** |  |  | 58.91 | **<0.001** |
| Yes | 1,195 (65.7) | 1,202 (53.8) |  |  |
| No | 624 (34.3) | 1,033 (46.2) |  |  |
