# Supplementary material for: Socio-economic inequalities in the breadth of internet use before and during the COVID-19 pandemic among older adults in England
Source: PLoS One. 2024 May 9;19(5):e0303061. doi: 10.1371/journal.pone.0303061 (PMC11081243; doi:10.1371/journal.pone.0303061)
Supplement: S4 Table — Note: n, number of participants. Bold denotes statistical significance (p<0.05). (DOCX) [file pone.0303061.s005.docx]

| **Online activities** | **Male participants**  ***n* (%)** | **Female participants**  ***n* (%)** | $\boldsymbol{\chi}$**^2^** | ***p* value** |
| --- | --- | --- | --- | --- |
| **Emails** |  |  | 2.81 | 0.093 |
| Yes | 1,566 (89.5) | 1,894 (87.8) |  |  |
| No | 184 (10.5) | 264 (12.2) |  |  |
| **Calls** |  |  | 22.40 | **<0.001** |
| Yes | 973 (55.6) | 1,361 (63.1) |  |  |
| No | 777 (44.4) | 797 (36.9) |  |  |
| **Health** |  |  | 6.97 | **0.008** |
| Yes | 697 (39.8) | 950 (44.0) |  |  |
| No | 1,053 (60.2) | 1,208 (56.0) |  |  |
| **Entertainment** |  |  | 0.02 | 0.897 |
| Yes | 787 (45.0) | 966 (44.8) |  |  |
| No | 963 (55.0) | 1,192 (55.2) |  |  |
| **News** |  |  | 28.19 | **<0.001** |
| Yes | 1,016 (58.1) | 1,069 (49.5) |  |  |
| No | 734 (41.9) | 1,089 (50.5) |  |  |
| **Market** |  |  | 8.43 | **0.004** |
| Yes | 1,298 (74.2) | 1,510 (70.0) |  |  |
| No | 452 (25.8) | 648 (30.0) |  |  |
| **Social networking** |  |  | 30.83 | **<0.001** |
| Yes | 662 (37.8) | 1,007 (46.7) |  |  |
| No | 1,088 (62.2) | 1,151 (53.3) |  |  |
| **Internet transactions** |  |  | 44.68 | **<0.001** |
| Yes | 1,101 (62.9) | 1,128 (52.3) |  |  |
| No | 649 (37.1) | 1,030 (47.7) |  |  |
