# Supplementary material for: Socio-economic inequalities in the breadth of internet use before and during the COVID-19 pandemic among older adults in England
Source: PLoS One. 2024 May 9;19(5):e0303061. doi: 10.1371/journal.pone.0303061 (PMC11081243; doi:10.1371/journal.pone.0303061)
Supplement: S5 Table — Note: AIC, Akaike Information Criterion; BIC, Bayesian Information Criterion; SSABIC, sample-size adjusted Bayesian Information Criterion; VLMR-LRT p, Vuong-Lo-Mendell-Rubin likelihood ratio test p-value; Adj. LMR-LRT p, Lo-Mendell-Rubin adjusted likelihood ratio test p-value. Bold font indicates the selected model. The class sizes are based on participants’ most likely latent class membership. (DOCX) [file pone.0303061.s006.docx]

|  | **AIC** | **BIC** | **SSABIC** | **Entropy** | **VLMR-LRT *p*** | **Adj. LMR-LRT *p*** | **Class size** |
| --- | --- | --- | --- | --- | --- | --- | --- |
| **Male participants** | | | | | | | |
| *Pre-pandemic (n=1,819)* | | | | | | | |
| 1 class | 16970.739 | 17014.788 | 16989.372 |  |  |  | 1,819 |
| 2 classes | 15562.702 | 15656.305 | 15602.296 | 0.698 | <0.001 | <0.001 | 738/1,081 |
| **3 classes** | **15466.243** | **15609.400** | **15526.800** | **0.566** | **0.002** | **0.002** | **733/681/405** |
| 4 classes | 15445.320 | 15638.031 | 15526.838 | 0.588 | 0.294 | 0.299 | 123/625/762/309 |
| 5 classes | 15433.150 | 15675.416 | 15535.630 | 0.620 | 0.487 | 0.490 | 145/852/446/160/216 |
| 6 classes | 15425.251 | 15717.071 | 15548.693 | 0.613 | 0.037 | 0.038 | 477/136/316/550/130/210 |
| *During COVID-19 (n=1,750)* | | | | | | | |
| 1 class | 17367.194 | 17410.933 | 17385.518 |  |  |  | 1,750 |
| 2 classes | 16346.860 | 16439.805 | 16385.797 | 0.635 | <0.001 | <0.001 | 763/987 |
| **3 classes** | **16227.823** | **16369.975** | **16287.375** | **0.575** | **<0.001** | **<0.001** | **550/301/899** |
| 4 classes | 16195.120 | 16386.478 | 16275.286 | 0.568 | 0.103 | 0.106 | 499/265/871/115 |
| 5 classes | 16173.974 | 16414.538 | 16274.754 | 0.613 | 0.017 | 0.018 | 310/697/142/431/170 |
| 6 classes | 16172.639 | 16462.410 | 16294.034 | 0.634 | 0.515 | 0.521 | 376/751/145/279/110/89 |
| **Female participants** | | | | | | | |
| *Pre-pandemic (n=2,235)* | | | | | | | |
| 1 class | 22129.547 | 22175.243 | 22149.826 |  |  |  | 2,235 |
| 2 classes | 20596.360 | 20693.464 | 20639.452 | 0.659 | <0.001 | <0.001 | 1,047/1,188 |
| **3 classes** | **20487.866** | **20636.378** | **20553.772** | **0.572** | **0.014** | **0.015** | **465/523/1,247** |
| 4 classes | 20420.669 | 20620.589 | 20509.389 | 0.525 | 0.308 | 0.312 | 479/586/617/553 |
| 5 classes | 20369.813 | 20621.141 | 20481.345 | 0.561 | 0.003 | 0.003 | 498/489/518/601/129 |
| 6 classes | 20354.502 | 20657.238 | 20488.849 | 0.547 | 0.757 | 0.759 | 162/523/414/431/517/188 |
| *During COVID-19 (n=2,158)* | | | | | | | |
| 1 class | 21988.854 | 22034.269 | 22008.852 |  |  |  | 2,158 |
| **2 classes** | **20762.620** | **20859.128** | **20805.117** | **0.647** | **<0.001** | **<0.001** | **884/1,274** |
| 3 classes | 20706.346 | 20853.947 | 20771.341 | 0.545 | 0.122 | 0.125 | 557/1,065/536 |
| 4 classes | 20654.493 | 20853.186 | 20741.987 | 0.571 | 0.002 | 0.002 | 459/197/755/747 |
| 5 classes | 20612.903 | 20862.688 | 20722.894 | 0.539 | 0.056 | 0.058 | 420/151/558/578/451 |
| 6 classes | 20606.125 | 20907.003 | 20738.615 | 0.601 | 0.173 | 0.177 | 558/582/155/177/385/301 |
