# Supplementary material for: Socio-economic inequalities in the breadth of internet use before and during the COVID-19 pandemic among older adults in England
Source: PLoS One. 2024 May 9;19(5):e0303061. doi: 10.1371/journal.pone.0303061 (PMC11081243; doi:10.1371/journal.pone.0303061)
Supplement: S6 Table — Note: n, number of participants; SD, standard deviation. aAge was collapsed to 90 for participants aged 90+ years. Demographic information was collected at baseline and fed-forward or updated in the COVID-19 sub-study. (DOCX) [file pone.0303061.s007.docx]

|  | **Pre-pandemic** | | | | **Intra-pandemic** | | | |
| --- | --- | --- | --- | --- | --- | --- | --- | --- |
|  | **Overall**  **(*n*=1,819)** | **Low**  **(*n*=405)** | **Medium**  **(*n*=681)** | **High**  **(*n*=733)** | **Overall**  **(*n*=1,750)** | **Low**  **(*n*=301)** | **Medium**  **(*n*=899)** | **High**  **(*n*=550)** |
| **Age, mean (SD)^a^** | 71.3 (6.9) | 73.7 (7.6) | 71.9 (6.9) | 69.3 (5.9) | 71.9 (6.8) | 73.9 (7.1) | 72.5 (6.8) | 69.7 (6.0) |
| **Age, *n* (%)** |  |  |  |  |  |  |  |  |
| 60-69 years | 800 (44.0) | 135 (33.3) | 270 (39.6) | 395 (53.9) | 709 (40.5) | 92 (30.6) | 327 (36.4) | 290 (52.7) |
| 70-79 years | 760 (41.8) | 169 (41.7) | 300 (44.1) | 291 (39.7) | 791 (45.2) | 145 (48.2) | 422 (46.9) | 224 (40.7) |
| 80+ years | 259 (14.2) | 101 (24.9) | 111 (16.3) | 47 (6.4) | 250 (14.3) | 64 (21.3) | 150 (16.7) | 36 (6.5) |
| Missing data | 0 (0.0) | 0 (0.0) | 0 (0.0) | 0 (0.0) | 0 (0.0) | 0 (0.0) | 0 (0.0) | 0 (0.0) |
| **Ethnicity, *n* (%)** |  |  |  |  |  |  |  |  |
| White | 1,773 (97.5) | 394 (97.3) | 661 (97.1) | 718 (98.0) | 1,700 (97.1) | 290 (96.3) | 874 (97.2) | 536 (97.5) |
| Non-White | 46 (2.5) | 11 (2.7) | 20 (2.9) | 15 (2.0) | 50 (2.9) | 11 (3.7) | 25 (2.8) | 14 (2.5) |
| Missing data | 0 (0.0) | 0 (0.0) | 0 (0.0) | 0 (0.0) | 0 (0.0) | 0 (0.0) | 0 (0.0) | 0 (0.0) |
| **Marital status, *n* (%)** |  |  |  |  |  |  |  |  |
| Single | 119 (6.5) | 27 (6.7) | 43 (6.3) | 49 (6.7) | 103 (5.9) | 22 (7.3) | 47 (5.2) | 34 (6.2) |
| Separated/divorced | 188 (10.3) | 53 (13.1) | 72 (10.6) | 63 (8.6) | 160 (9.1) | 39 (13.0) | 77 (8.6) | 44 (8.0) |
| Widowed | 142 (7.8) | 45 (11.1) | 58 (8.5) | 39 (5.3) | 114 (6.5) | 29 (9.6) | 58 (6.5) | 27 (4.9) |
| Married/with a partner | 1,162 (63.9) | 247 (61.0) | 427 (62.7) | 488 (66.6) | 1,136 (64.9) | 184 (61.1) | 601 (66.9) | 351 (63.8) |
| Remarried | 207 (11.4) | 33 (8.1) | 81 (11.9) | 93 (12.7) | 188 (10.7) | 24 (8.0) | 98 (10.9) | 66 (12.0) |
| Missing data | 1 (0.1) | 0 (0.0) | 0 (0.0) | 1 (0.1) | 49 (2.8) | 3 (1.0) | 18 (2.0) | 28 (5.1) |
| **Employment, *n* (%)** |  |  |  |  |  |  |  |  |
| Retired | 1,351 (74.3) | 314 (77.5) | 511 (75.0) | 526 (71.8) | 1,366 (78.1) | 237 (78.7) | 718 (79.9) | 411 (74.7) |
| Unemployed | 6 (0.3) | 1 (0.2) | 3 (0.4) | 2 (0.3) | 12 (0.7) | 3 (1.0) | 6 (0.7) | 3 (0.5) |
| Permanently sick or disabled | 31 (1.7) | 13 (3.2) | 14 (2.1) | 4 (0.5) | 20 (1.1) | 6 (2.0) | 8 (0.9) | 6 (1.1) |
| Looking after home or family | 17 (0.9) | 3 (0.7) | 5 (0.7) | 9 (1.2) | 15 (0.9) | 1 (0.3) | 8 (0.9) | 6 (1.1) |
| Semi-retired | 28 (1.5) | 4 (1.0) | 8 (1.2) | 16 (2.2) | – | – | – | – |
| Employed | 248 (13.6) | 43 (10.6) | 82 (12.0) | 123 (16.8) | 164 (9.4) | 26 (8.6) | 70 (7.8) | 68 (12.4) |
| Self-employed | 136 (7.5) | 25 (6.2) | 58 (8.5) | 53 (7.2) |  |  |  |  |
| And currently working | – | – | – | – | 79 (4.5) | 13 (4.3) | 47 (5.2) | 19 (3.5) |
| But not currently working | – | – | – | – | 41 (2.3) | 3 (1.0) | 16 (1.8) | 22 (4.0) |
| Paid/unpaid leave from employment (including furlough) | – | – | – | – | 53 (3.0) | 12 (4.0) | 26 (2.9) | 15 (2.7) |
| Missing data | 2 (0.1) | 2 (0.5) | 0 (0.0) | 0 (0.0) | 0 (0.0) | 0 (0.0) | 0 (0.0) | 0 (0.0) |
| **Living status, *n* (%)** |  |  |  |  |  |  |  |  |
| Living alone | 332 (18.3) | 96 (23.7) | 134 (19.7) | 102 (13.9) | 303 (17.3) | 63 (20.9) | 157 (17.5) | 83 (15.1) |
| Not living alone | 1,487 (81.7) | 309 (76.3) | 547 (80.3) | 631 (86.1) | 1,434 (81.9) | 235 (78.1) | 735 (81.8) | 464 (84.4) |
| Missing data | 0 (0.0) | 0 (0.0) | 0 (0.0) | 0 (0.0) | 13 (0.7) | 3 (1.0) | 7 (0.8) | 3 (0.5) |
| **Limiting long-standing illness, disability, or infirmity, *n* (%)** |  |  |  |  |  |  |  |  |
| No | 1,244 (68.4) | 251 (62.0) | 443 (65.1) | 550 (75.0) | 1,247 (71.3) | 202 (67.1) | 626 (69.6) | 419 (76.2) |
| Yes | 570 (31.3) | 154 (38.0) | 234 (34.4) | 182 (24.8) | 503 (28.7) | 99 (32.9) | 273 (30.4) | 131 (23.8) |
| Missing data | 5 (0.3) | 0 (0.0) | 4 (0.6) | 1 (0.1) | 0 (0.0) | 0 (0.0) | 0 (0.0) | 0 (0.0) |
| **Education, *n* (%)** |  |  |  |  |  |  |  |  |
| Low | 152 (8.4) | 73 (18.0) | 50 (7.3) | 29 (4.0) | 114 (6.5) | 43 (14.3) | 51 (5.7) | 20 (3.6) |
| Medium | 565 (31.1) | 161 (39.8) | 235 (34.5) | 169 (23.1) | 505 (28.9) | 125 (41.5) | 269 (29.9) | 111 (20.2) |
| High | 1,019 (56.0) | 143 (35.3) | 364 (53.5) | 512 (69.8) | 949 (54.2) | 102 (33.9) | 496 (55.2) | 351 (63.8) |
| Missing data | 83 (4.6) | 28 (6.9) | 32 (4.7) | 23 (3.1) | 182 (10.4) | 31 (10.3) | 83 (9.2) | 68 (12.4) |
| **Occupational class, *n* (%)** |  |  |  |  |  |  |  |  |
| Routine and manual | 502 (27.6) | 176 (43.5) | 180 (26.4) | 146 (19.9) | 421 (24.1) | 120 (39.9) | 223 (24.8) | 78 (14.2) |
| Intermediate | 377 (20.7) | 93 (23.0) | 157 (23.1) | 127 (17.3) | 350 (20.0) | 73 (24.3) | 176 (19.6) | 101 (18.4) |
| Higher managerial, administrative and professional | 857 (47.1) | 125 (30.9) | 310 (45.5) | 422 (57.6) | 789 (45.1) | 85 (28.2) | 418 (46.5) | 286 (52.0) |
| Missing data | 83 (4.6) | 11 (2.7) | 34 (5.0) | 38 (5.2) | 190 (10.9) | 23 (7.6) | 82 (9.1) | 85 (15.5) |
| **Wealth, *n* (%)** |  |  |  |  |  |  |  |  |
| 1^st^ quintile (lowest) | 164 (9.0) | 56 (13.8) | 65 (9.5) | 43 (5.9) | 139 (7.9) | 42 (14.0) | 75 (8.3) | 22 (4.0) |
| 2^nd^ quintile | 223 (12.3) | 73 (18.0) | 88 (12.9) | 62 (8.5) | 186 (10.6) | 44 (14.6) | 95 (10.6) | 47 (8.5) |
| 3^rd^ quintile | 381 (20.9) | 114 (28.1) | 144 (21.1) | 123 (16.8) | 332 (19.0) | 75 (24.9) | 179 (19.9) | 78 (14.2) |
| 4^th^ quintile | 482 (26.5) | 103 (25.4) | 174 (25.6) | 205 (28.0) | 441 (25.2) | 76 (25.2) | 231 (25.7) | 134 (24.4) |
| 5^th^ quintile (highest) | 537 (29.5) | 55 (13.6) | 205 (30.1) | 277 (37.8) | 515 (29.4) | 48 (15.9) | 264 (29.4) | 203 (36.9) |
| Missing data | 32 (1.8) | 4 (1.0) | 5 (0.7) | 23 (3.1) | 137 (7.8) | 16 (5.3) | 55 (6.1) | 66 (12.0) |
| **Internet frequency, *n* (%)** |  |  |  |  |  |  |  |  |
| Low frequency | 84 (4.6) | 75 (18.5) | 8 (1.2) | 1 (0.1) | 29 (1.7) | 22 (7.3) | 7 (0.8) | 0 (0.0) |
| Moderate frequency | 208 (11.4) | 132 (32.6) | 69 (10.1) | 7 (1.0) | 148 (8.5) | 88 (29.2) | 56 (6.2) | 4 (0.7) |
| High frequency | 1,527 (83.9) | 198 (48.9) | 604 (88.7) | 725 (98.9) | 1,573 (89.9) | 191 (63.5) | 836 (93.0) | 546 (99.3) |
| Missing data | 0 (0.0) | 0 (0.0) | 0 (0.0) | 0 (0.0) | 0 (0.0) | 0 (0.0) | 0 (0.0) | 0 (0.0) |
