# Supplementary material for: Socio-economic inequalities in the breadth of internet use before and during the COVID-19 pandemic among older adults in England
Source: PLoS One. 2024 May 9;19(5):e0303061. doi: 10.1371/journal.pone.0303061 (PMC11081243; doi:10.1371/journal.pone.0303061)
Supplement: S7 Table — Note: n, number of participants; SD, standard deviation. aAge was collapsed to 90 for participants aged 90+ years. Demographic information was collected at baseline and fed-forward or updated in the COVID-19 sub-study. (DOCX) [file pone.0303061.s008.docx]

|  | **Pre-pandemic** | | | | **Intra-pandemic** | | |
| --- | --- | --- | --- | --- | --- | --- | --- |
|  | **Overall (*n*=2,235)** | **Low**  **(*n*=523)** | **Medium**  **(*n*=1,247)** | **High**  **(*n*=465)** | **Overall (*n*=2,158)** | **Low**  **(*n*=1,274)** | **High**  **(*n*=884)** |
| **Age, mean (SD)^a^** | 70.8 (6.7) | 73.6 (7.4) | 70.9 (6.3) | 67.6 (5.4) | 71.5 (6.7) | 72.9 (7.0) | 69.5 (5.8) |
| **Age, *n* (%)** |  |  |  |  |  |  |  |
| 60-69 years | 1,043 (46.7) | 172 (32.9) | 552 (44.3) | 319 (68.6) | 926 (42.9) | 449 (35.2) | 477 (54.0) |
| 70-79 years | 907 (40.6) | 219 (41.9) | 557 (44.7) | 131 (28.2) | 937 (43.4) | 582 (45.7) | 355 (40.2) |
| 80+ years | 285 (12.8) | 132 (25.2) | 138 (11.1) | 15 (3.2) | 295 (13.7) | 243 (19.1) | 52 (5.9) |
| Missing data | 0 (0.0) | 0 (0.0) | 0 (0.0) | 0 (0.0) | 0 (0.0) | 0 (0.0) | 0 (0.0) |
| **Ethnicity, *n* (%)** |  |  |  |  |  |  |  |
| White | 2,180 (97.5) | 507 (96.9) | 1,217 (97.6) | 456 (98.1) | 2,095 (97.1) | 1,225 (96.2) | 870 (98.4) |
| Non-White | 55 (2.5) | 16 (3.1) | 30 (2.4) | 9 (1.9) | 63 (2.9) | 49 (3.8) | 14 (1.6) |
| Missing data | 0 (0.0) | 0 (0.0) | 0 (0.0) | 0 (0.0) | 0 (0.0) | 0 (0.0) | 0 (0.0) |
| **Marital status, *n* (%)** |  |  |  |  |  |  |  |
| Single | 116 (5.2) | 19 (3.6) | 69 (5.5) | 28 (6.0) | 114 (5.3) | 65 (5.1) | 49 (5.5) |
| Separated/divorced | 355 (15.9) | 85 (16.3) | 184 (14.8) | 86 (18.5) | 317 (14.7) | 171 (13.4) | 146 (16.5) |
| Widowed | 431 (19.3) | 136 (26.0) | 234 (18.8) | 61 (13.1) | 369 (17.1) | 257 (20.2) | 112 (12.7) |
| Married/with a partner | 1,114 (49.8) | 230 (44.0) | 646 (51.8) | 238 (51.2) | 1,108 (51.3) | 648 (50.9) | 460 (52.0) |
| Remarried | 219 (9.8) | 53 (10.1) | 114 (9.1) | 52 (11.2) | 194 (9.0) | 107 (8.4) | 87 (9.8) |
| Missing data | 0 (0.0) | 0 (0.0) | 0 (0.0) | 0 (0.0) | 56 (2.6) | 26 (2.0) | 30 (3.4) |
| **Employment, *n* (%)** |  |  |  |  |  |  |  |
| Retired | 1,711 (76.6) | 423 (80.9) | 968 (77.6) | 320 (68.8) | 1,674 (77.6) | 1,007 (79.0) | 667 (75.5) |
| Unemployed | 9 (0.4) | 3 (0.6) | 2 (0.2) | 4 (0.9) | 16 (0.7) | 11 (0.9) | 5 (0.6) |
| Permanently sick or disabled | 48 (2.1) | 16 (3.1) | 27 (2.2) | 5 (1.1) | 48 (2.2) | 34 (2.7) | 14 (1.6) |
| Looking after home or family | 103 (4.6) | 25 (4.8) | 65 (5.2) | 13 (2.8) | 94 (4.4) | 62 (4.9) | 32 (3.6) |
| Semi-retired | 14 (0.6) | 2 (0.4) | 6 (0.5) | 6 (1.3) | – | – | – |
| Employed | 265 (11.9) | 45 (8.6) | 135 (10.8) | 85 (18.3) | 170 (7.9) | 85 (6.7) | 85 (9.6) |
| Self-employed | 78 (3.5) | 9 (1.7) | 38 (3.0) | 31 (6.7) |  |  |  |
| And currently working | – | – | – | – | 40 (1.9) | 15 (1.2) | 25 (2.8) |
| But not currently working | – | – | – | – | 31 (1.4) | 16 (1.3) | 15 (1.7) |
| Paid/unpaid leave from employment (including furlough) | – | – | – | – | 84 (3.9) | 43 (3.4) | 41 (4.6) |
| Missing data | 7 (0.3) | 0 (0.0) | 6 (0.5) | 1 (0.2) | 1 (0.0) | 1 (0.1) | 0 (0.0) |
| **Living status, *n* (%)** |  |  |  |  |  |  |  |
| Living alone | 668 (29.9) | 189 (36.1) | 358 (28.7) | 121 (26.0) | 659 (30.5) | 412 (32.3) | 247 (27.9) |
| Not living alone | 1,566 (70.1) | 334 (63.9) | 888 (71.2) | 344 (74.0) | 1,489 (69.0) | 856 (67.2) | 633 (71.6) |
| Missing data | 1 (0.0) | 0 (0.0) | 1 (0.1) | 0 (0.0) | 10 (0.5) | 6 (0.5) | 4 (0.5) |
| **Limiting long-standing illness, disability, or infirmity, *n* (%)** |  |  |  |  |  |  |  |
| No | 1,442 (64.5) | 301 (57.6) | 819 (65.7) | 322 (69.2) | 1,449 (67.1) | 829 (65.1) | 620 (70.1) |
| Yes | 791 (35.4) | 221 (42.3) | 427 (34.2) | 143 (30.8) | 708 (32.8) | 444 (34.9) | 264 (29.9) |
| Missing data | 2 (0.1) | 1 (0.2) | 1 (0.1) | 0 (0.0) | 1 (0.0) | 1 (0.1) | 0 (0.0) |
| **Education, *n* (%)** |  |  |  |  |  |  |  |
| Low | 319 (14.3) | 143 (27.3) | 146 (11.7) | 30 (6.5) | 260 (12.0) | 207 (16.2) | 53 (6.0) |
| Medium | 838 (37.5) | 197 (37.7) | 483 (38.7) | 158 (34.0) | 770 (35.7) | 480 (37.7) | 290 (32.8) |
| High | 864 (38.7) | 120 (22.9) | 493 (39.5) | 251 (54.0) | 810 (37.5) | 375 (29.4) | 435 (49.2) |
| Missing data | 214 (9.6) | 63 (12.0) | 125 (10.0) | 26 (5.6) | 318 (14.7) | 212 (16.6) | 106 (12.0) |
| **Occupational class, *n* (%)** |  |  |  |  |  |  |  |
| Routine and manual | 641 (28.7) | 234 (44.7) | 339 (27.2) | 68 (14.6) | 549 (25.4) | 400 (31.4) | 149 (16.9) |
| Intermediate | 693 (31.0) | 155 (29.6) | 401 (32.2) | 137 (29.5) | 645 (29.9) | 387 (30.4) | 258 (29.2) |
| Higher managerial, administrative and professional | 756 (33.8) | 101 (19.3) | 431 (34.6) | 224 (48.2) | 696 (32.3) | 334 (26.2) | 362 (41.0) |
| Missing data | 145 (6.5) | 33 (6.3) | 76 (6.1) | 36 (7.7) | 268 (12.4) | 153 (12.0) | 115 (13.0) |
| **Wealth, *n* (%)** |  |  |  |  |  |  |  |
| 1^st^ quintile (lowest) | 264 (11.8) | 96 (18.4) | 137 (11.0) | 31 (6.7) | 214 (9.9) | 157 (12.3) | 57 (6.4) |
| 2^nd^ quintile | 338 (15.1) | 128 (24.5) | 174 (14.0) | 36 (7.7) | 293 (13.6) | 220 (17.3) | 73 (8.3) |
| 3^rd^ quintile | 476 (21.3) | 114 (21.8) | 267 (21.4) | 95 (20.4) | 441 (20.4) | 278 (21.8) | 163 (18.4) |
| 4^th^ quintile | 544 (24.3) | 105 (20.1) | 321 (25.7) | 118 (25.4) | 511 (23.7) | 286 (22.4) | 225 (25.5) |
| 5^th^ quintile (highest) | 586 (26.2) | 75 (14.3) | 333 (26.7) | 178 (38.3) | 545 (25.3) | 251 (19.7) | 294 (33.3) |
| Missing data | 27 (1.2) | 5 (1.0) | 15 (1.2) | 7 (1.5) | 154 (7.1) | 82 (6.4) | 72 (8.1) |
| **Internet frequency, *n* (%)** |  |  |  |  |  |  |  |
| Low frequency | 180 (8.1) | 153 (29.3) | 27 (2.2) | 0 (0.0) | 65 (3.0) | 65 (5.1) | 0 (0.0) |
| Moderate frequency | 335 (15.0) | 162 (31.0) | 168 (13.5) | 5 (1.1) | 246 (11.4) | 231 (18.1) | 15 (1.7) |
| High frequency | 1,720 (77.0) | 208 (39.8) | 1,052 (84.4) | 460 (98.9) | 1,847 (85.6) | 978 (76.8) | 869 (98.3) |
| Missing data | 0 (0.0) | 0 (0.0) | 0 (0.0) | 0 (0.0) | 0 (0.0) | 0 (0.0) | 0 (0.0) |
