# Supplementary material for: Socio-economic inequalities in the breadth of internet use before and during the COVID-19 pandemic among older adults in England
Source: PLoS One. 2024 May 9;19(5):e0303061. doi: 10.1371/journal.pone.0303061 (PMC11081243; doi:10.1371/journal.pone.0303061)
Supplement: S8 Table — Note: LCA, latent class analysis. The proportions (%) are based on participants’ most likely latent class membership. (DOCX) [file pone.0303061.s009.docx]

|  | **During COVID-19** | | |
| --- | --- | --- | --- |
| **Pre-pandemic** | Low class (16.3%) | Medium class (52.7%) | High class (30.9%) |
| *Male participants (n=1,506)* |  |  |  |
| Low class (17.3%) | 0.623 | 0.354 | 0.023 |
| Medium class (37.8%) | 0.111 | 0.732 | 0.158 |
| High class (44.9%) | 0.031 | 0.422 | 0.547 |
|  | Low class (58.5%) |  | High class (41.5%) |
| *Female participants (n=1,855)* |  | | |
| Low class (16.8%) | 0.945 | – | 0.055 |
| Medium class (59.4%) | 0.640 | – | 0.360 |
| High class (23.9%) | 0.196 | – | 0.804 |
