# Supplementary material for: Socio-economic inequalities in the breadth of internet use before and during the COVID-19 pandemic among older adults in England
Source: PLoS One. 2024 May 9;19(5):e0303061. doi: 10.1371/journal.pone.0303061 (PMC11081243; doi:10.1371/journal.pone.0303061)
Supplement: S9 Table — Note: LTA, latent transition analysis; AIC, Akaike Information Criterion; BIC, Bayesian Information Criterion; SSABIC, sample-size adjusted Bayesian Information Criterion. (DOCX) [file pone.0303061.s010.docx]

|  | **AIC** | **BIC** | **SSABIC** | **Entropy** |
| --- | --- | --- | --- | --- |
| *Male participants* |  |  |  |  |
| Unconditional (*n*=2,063) | 30644.386 | 30959.773 | 30781.856 | 0.850 |
| Conditional (*n*=1,733) | 26213.589 | 26617.452 | 26382.361 | 0.853 |
| *Female participants* |  |  |  |  |
| Unconditional (*n*=2,538) | 40340.496 | 40603.257 | 40460.280 | 0.756 |
| Conditional (*n*=2,008) | 32043.565 | 32396.674 | 32196.519 | 0.774 |
