# Supplementary material for: Socio-economic inequalities in the breadth of internet use before and during the COVID-19 pandemic among older adults in England
Source: PLoS One. 2024 May 9;19(5):e0303061. doi: 10.1371/journal.pone.0303061 (PMC11081243; doi:10.1371/journal.pone.0303061)
Supplement: S10 Table — Note: LTA, latent transition analysis. The proportions (%) are based on participants’ most likely latent class pattern. The latent transition probabilities are based on the estimated model. (DOCX) [file pone.0303061.s011.docx]

|  | **During COVID-19** | | |
| --- | --- | --- | --- |
| **Pre-pandemic** | Low class (25.8%) | Medium class (42.9%) | High class (31.3%) |
| *Male participants (n=1,733)* |  |  |  |
| Low class (25.9%) | 0.996 | 0.000 | 0.004 |
| Medium class (42.3%) | 0.000 | 1.000 | 0.000 |
| High class (31.9%) | 0.001 | 0.016 | 0.983 |
|  | Low class (58.7%) |  | High class (41.3%) |
| *Female participants (n=2,008)* |  | | |
| Low class (24.8%) | 1.000 | – | 0.000 |
| Medium class (33.7%) | 0.967 | – | 0.033 |
| High class (41.5%) | 0.020 | – | 0.980 |
