# Supplementary material for: Socio-economic inequalities in the breadth of internet use before and during the COVID-19 pandemic among older adults in England
Source: PLoS One. 2024 May 9;19(5):e0303061. doi: 10.1371/journal.pone.0303061 (PMC11081243; doi:10.1371/journal.pone.0303061)
Supplement: S11 Table — Note: LTA, latent transition analysis; SE, standard error; OR, odds ratio; CI, confidence intervals. The counts (n) are based on participants’ most likely latent class pattern. Bold denotes statistical significance (p<0.05). (DOCX) [file pone.0303061.s012.docx]

|  | **Estimate (SE)** | ***p*** | **OR (95% CI)** |
| --- | --- | --- | --- |
| **Male participants (*n*=1,733)** |  |  |  |
| Medium class (*n*=733) |  |  |  |
| Age | -0.044 (0.021) | **0.036** | 0.957 (0.919, 0.997) |
| Education |  |  |  |
| Low (reference) |  |  |  |
| Medium | 0.983 (0.332) | **0.003** | 2.672 (1.393, 5.126) |
| High | 1.678 (0.338) | **<0.001** | 5.356 (2.763, 10.381) |
| Occupational class |  |  |  |
| Routine and manual (reference) |  |  |  |
| Intermediate | -0.094 (0.259) | 0.715 | 0.910 (0.548, 1.511) |
| Higher managerial, administrative and professional | 0.509 (0.237) | **0.032** | 1.664 (1.045, 2.649) |
| Wealth |  |  |  |
| 1^st^ quintile (reference) |  |  |  |
| 2^nd^ quintile | -0.149 (0.339) | 0.660 | 0.862 (0.444, 1.673) |
| 3^rd^ quintile | -0.221 (0.291) | 0.448 | 0.802 (0.454, 1.418) |
| 4^th^ quintile | 0.000 (0.324) | 1.000 | 1.000 (0.530, 1.886) |
| 5^th^ quintile (highest) | 0.922 (0.341) | **0.007** | 2.515 (1.288, 4.909) |
| High class (*n*=552) |  |  |  |
| Age | -0.179 (0.025) | **<0.001** | 0.836 (0.796, 0.878) |
| Education |  |  |  |
| Low (reference) |  |  |  |
| Medium | 0.243 (0.378) | 0.520 | 1.275 (0.608, 2.673) |
| High | 1.373 (0.419) | **0.001** | 3.946 (1.736, 8.970) |
| Occupational class |  |  |  |
| Routine and manual (reference) |  |  |  |
| Intermediate | 0.501 (0.276) | 0.069 | 1.650 (0.961, 2.832) |
| Higher managerial, administrative and professional | 1.428 (0.290) | **<0.001** | 4.172 (2.361, 7.373) |
| Wealth |  |  |  |
| 1^st^ quintile (reference) |  |  |  |
| 2^nd^ quintile | 0.845 (0.486) | 0.082 | 2.327 (0.899, 6.027) |
| 3^rd^ quintile | 0.896 (0.485) | 0.065 | 2.449 (0.946, 6.338) |
| 4^th^ quintile | 1.464 (0.507) | **0.004** | 4.322 (1.599, 11.680) |
| 5^th^ quintile (highest) | 2.630 (0.572) | **<0.001** | 13.871 (4.520, 42.566) |
| **Female participants (*n*=2,008)** |  |  |  |
| Medium class (*n*=676) |  |  |  |
| Age | -0.032 (0.033) | 0.339 | 0.969 (0.908, 1.034) |
| Education |  |  |  |
| Low (reference) |  |  |  |
| Medium | 1.373 (0.416) | **0.001** | 3.946 (1.745, 8.926) |
| High | 1.588 (0.516) | **0.002** | 4.894 (1.781, 13.452) |
| Occupational class |  |  |  |
| Routine and manual (reference) |  |  |  |
| Intermediate | 0.882 (0.279) | **0.002** | 2.415 (1.399, 4.169) |
| Higher managerial, administrative and professional | 1.495 (0.331) | **<0.001** | 4.461 (2.334, 8.529) |
| Wealth |  |  |  |
| 1^st^ quintile (reference) |  |  |  |
| 2^nd^ quintile | 0.035 (0.404) | 0.930 | 1.036 (0.469, 2.287) |
| 3^rd^ quintile | 0.678 (0.426) | 0.111 | 1.971 (0.856, 4.539) |
| 4^th^ quintile | 0.779 (0.487) | 0.110 | 2.179 (0.838, 5.664) |
| 5^th^ quintile (highest) | 1.184 (0.519) | **0.023** | 3.266 (1.180, 9.041) |
| High class (*n*=834) |  |  |  |
| Age | -0.170 (0.028) | **<0.001** | 0.844 (0.798, 0.892) |
| Education |  |  |  |
| Low (reference) |  |  |  |
| Medium | 1.188 (0.261) | **<0.001** | 3.282 (1.966, 5.479) |
| High | 1.773 (0.304) | **<0.001** | 5.889 (3.246, 10.683) |
| Occupational class |  |  |  |
| Routine and manual (reference) |  |  |  |
| Intermediate | 1.130 (0.222) | **<0.001** | 3.096 (2.004, 4.783) |
| Higher managerial, administrative and professional | 2.033 (0.292) | **<0.001** | 7.638 (4.313, 13.527) |
| Wealth |  |  |  |
| 1^st^ quintile (reference) |  |  |  |
| 2^nd^ quintile | -0.022 (0.319) | 0.944 | 0.978 (0.523, 1.828) |
| 3^rd^ quintile | 1.134 (0.320) | **<0.001** | 3.107 (1.659, 5.820) |
| 4^th^ quintile | 1.118 (0.320) | **<0.001** | 3.060 (1.633, 5.732) |
| 5^th^ quintile (highest) | 1.914 (0.360) | **<0.001** | 6.779 (3.347, 13.730) |
