# Supplementary material for: Socio-economic inequalities in the breadth of internet use before and during the COVID-19 pandemic among older adults in England
Source: PLoS One. 2024 May 9;19(5):e0303061. doi: 10.1371/journal.pone.0303061 (PMC11081243; doi:10.1371/journal.pone.0303061)
Supplement: Attachment — Submitted filename: Comments to the Authors PLOS.docx [file pone.0303061.s013.docx]

# Comments to the Authors

21.01.2024

| **line** | **comment** |
| --- | --- |
| 26 & 50 | Abstract & Introduction   - The literature review is rather thin. I’m aware that this paper uses an explorative approach, however, to elaborate the value of your study you need to provide a sufficient overview over existing literature - The research gap and the studies contribution to the field could be strengthened |
| abstract | A high degree of stability in latent class membership was observed over **time.However**, men experienced a stark decrease in online health information-**seeking.Our** results highlight that inequality regarding the range of functional and social opportunities provided by the internet prevailed during the pandemic.   - Please check if you forgot some spacings |
| 27 | “Understanding digital exclusion in vulnerable populations, such as older adults, during the COVID-19 pandemic could help tailor responses to future outbreaks.”   - Please pay attention avoiding ageist language (older adults != vulnerable). |
| 28 | (also abstract and keywords)   - Are you really doing a cohort study? (no control group) |
| 60 | Older adults are stereotypically described as a technology-resistant, homogeneous group of individuals [4,10].   - Please check if you cited the papers correctly. Do the papers provide empirical evidence for older adults are stereotypically described as a technology-resistant homogeneous group? I don’t think so. It seems like the studies cited use variations of this statement in their introductions, to build their study on a general discussion.   If you want to refer to this general statement, you do not need a text reference.  If you want to use a reference either, it needs to be something like a discourse analysis that finds this prescription of older adults in media / public discussion / … |
| 76 | Although a sole emphasis on digital means of social interaction could perpetuate ageism [15,16], the vital role of, and the projected increased reliance on, the internet during and beyond the COVID-19 pandemic may disadvantage older citizens who are offline or not conversant in contemporary technologies [9,23].   - The argument (digital means of social interaction perpetuates ageism) is not clear/intuitively. Please elaborate your argumentation. |
| 83 | To date, most work has focused on correlates and outcomes of internet access [6], whereas research delving into the specific activities for which the internet is used among older adults is more limited, despite some evidence of differential uptake of internet behaviours relative to younger age groups [25].   - Please investigate literature again. Thera are plenty of studies investigating different purposes of internet use… |
| 95 | which accelerated the digitalisation of many services.   - Consider removing “of many services”. Then your statement is more openly formulated. |
| 102 | A recent study explored differences in internet usage patterns among adults (aged 18-70+) in 104 the Netherlands during the COVID-19 pandemic [31]. Education, but not economic resources, was positively associated with information and communication uses [31]. Nonetheless, previous work has shown that income is particularly important in explaining occupational and consumptive uses [27,32], which were not assessed. As such, the influence of the COVID-19 pandemic on widening or reducing inequalities across a broader range of online activities remains unknown.   - Please revise your argumentation and read the literature more carefully (which other control variables are used in that study? Do you think income is similar to economic resources / other “socio-economic variables…?) - Please make clear what you mean by “socio-economic” aspects in your study? What are you looking at? Explain why you think different variables are related to the same concept and what makes them distinct to each other - Please check if the studies you cite provide evidence for the directionality of associations and check if COVID-19 is evidenced as cause of an effect |
| 119 | **Study design and participants**   - Both surveys underlying the analyses of this manuscript belonging to ELSA. However, even if the surveys are very similar and similar or identical questions are asked of the same individuals at different times (2018/19 and June/July 2020), there are still differences that can influence the results of the study. In addition to differences in the formulation of the questions and the response categories, the change in the survey mode (from CAPI to Online Survey or CATI) certainly poses the greatest challenge for the present study (that is interested in internet-use).   Perhaps there is already a publication that examines the mode effects of the ELSA surveys (or similar surveys) and can be referred to here? Moreover, the differences between the surveys and possible effects on the study could be discussed in more detail (in the study design section or in limitations section). |
| 123 | This sentence may be deleted: “The authors did not have access to personal data.”   - Information could be placed in the “Additional Information”-section |
| 131 | Select**ed ?** |
| 139 | If a definition of the "core members" of the ELSA survey is teased (because relevant for your article), please include it and not just make a reference to the documentation. |
| 147 | **Measures** |
| 150 | What time reference is used in the question on average frequency of internet or email usage? Please add. |
| 154 | For a better overview, the comparison of the response categories of the internet-use-questions of ELSA 2018/19 and ELSA COVID 2020 could be displayed reader-friendly in a table (like you did in table 1). |
| 158 | From lines 153 to 155 and table S1 it becomes clear, that answer categories are similar, but differ between the surveys regarding the recorded units of frequency, the number of categories, wording and the examples given.  For example, …  … your generated category “High” comprises individuals that responded “more than one a day” in one survey and “every day, or almost every day”  …your generated category “internet transactions” that comprises the answer categories “finances” and “public services” in the ELSA 2018/19 survey, but only “finances” in the ELSA COVID-19 survey.  Please point out these differences and possible consequences for the results of this study in the text. |
| 181 | Preliminary analyses explored differences between men and women across the eight types of internet activities, using Pearson’s chi-squared (𝜒2) test (S2 and S3 Tables).   - Please be precise: not all types of internet activities |
| 236 | Please check and update your link. It doesn’t seem to work properly:  <https://github.com/OliviaMalkowski/Inequalities-Internet-COVID-19.git> |
| 237 | Results   - You could list the characteristics of the identified classes in a table. This would make it easier for the reader to grasp the classes along with their |
|  | Discussion   - Please elaborate more on why the identification of sub-groups of internet users is relevant in the context of COVID-19 - The finding that health information seeking has decreased among in the COVID-19 wave in comparison to the pre-pandemic level is surprising and interesting. You discuss some plausible explanations. Another explanation could be the overburdening of the healthcare system and postponement of operations and treatments or the absence of medical examinations. Individuals may seek health information on the internet before undergoing surgery or treatment or after receiving diagnoses. - Please consider that not all older individuals may wish to increase their use of the internet (for various purposes). Differences in usage frequency do not necessarily stem from external barriers. To ensure the societal inclusion of all groups of older people, offerings and services should always be available in both digital and analog formats. |
|  | Literature |
| 551 | Please check this reference. It’s a preprint. Results are not presented. Please consider using another reference.  Kebede AS, Ozolins LL, Holst H, Galvin K. The digital engagement of older people: systematic scoping review protocol. JMIR Res Protoc. 2021 Jul 5;10(7):e25616. |
|  | Appendix |
| S1 Appendix | The table is not intuitively to understand, as some information is missing for the reader:   - Please provide a column header for col. 1. - Please add a note that the text written in bold letters in the “recommendation”-column are **added by you** and are referring to the section in the manuscript that covers each respective recommendation of the STROBE guidelines. OR: add an additional column containing references to your text (Item No.-column could be dropped, if there are formatting issues) - Please provide the source of the STROBE table as a note below the table. - Please provide an explanation of what the asterisk indicates. - Please provide a brief description of the STROBE guidelines/statement as a note below the table or in the text. |
| Table 1 | Please add information on the precise formulation of the questions in both surveys (in the “notes” below the table).  Please harmonize the wording (“variable” in the title; “item” in the column headers; …“response options” in the text). |
| S2 & S3 | In addition, please provide information on your data source in the table notes. |
| Fig 1 | The resolution could be improved to make the figure looking more professional. |
