# Supplementary material for: Socio-economic inequalities in the breadth of internet use before and during the COVID-19 pandemic among older adults in England
Source: PLoS One. 2024 May 9;19(5):e0303061. doi: 10.1371/journal.pone.0303061 (PMC11081243; doi:10.1371/journal.pone.0303061)
Supplement: Attachment — Submitted filename: Response to Reviewers.docx [file pone.0303061.s014.docx]

Dear Editor,

Thank you for the opportunity to revise the manuscript titled “Socio-economic inequalities in the breadth of internet use before and during the COVID-19 pandemic among older adults in England”. We appreciate the reviewers’ detailed suggestions to improve the quality of the manuscript. To address feedback on the literature section, we have added further detail to the introduction, ensuring that appropriate references are used that are relevant for our arguments. Furthermore, we have taken on board the reviewers’ feedback regarding the clarity of the internet use measures and have included an additional table in the Supplementary Material to enable a closer comparison of the survey questions and response options across the two waves (baseline and follow-up) of interest. We have added more information on the state of current knowledge and the contribution of this study in the introduction and discussion sections. We agree with the reviewers’ suggestion to expand on the limitations in the discussion section and have commented on the possible implications of changes in the mode of data collection and survey measures across timepoints. In addition, we have provided some further plausible explanations for the findings in the discussion section, particularly with regards to the observed decline in internet usage for health-related information. Moreover, we have improved the overall clarity of the manuscript and the tables/figures (including the Supplementary Material).

Journal Requirements:

When submitting your revision, we need you to address these additional requirements.

1. Please ensure that your manuscript meets PLOS ONE's style requirements, including those for file naming. The PLOS ONE style templates can be found at 
[https://journals.plos.org/plosone/s/file?id=wjVg/PLOSOne_formatting_sample_main_body.pdf](https://eur01.safelinks.protection.outlook.com/?url=https%3A%2F%2Fjournals.plos.org%2Fplosone%2Fs%2Ffile%3Fid%3DwjVg%2FPLOSOne_formatting_sample_main_body.pdf&data=05%7C02%7Cosm25%40bath.ac.uk%7Cfb6909f2e9014de0667408dc1cbff08c%7C377e3d224ea1422db0ad8fcc89406b9e%7C0%7C0%7C638416858198387983%7CUnknown%7CTWFpbGZsb3d8eyJWIjoiMC4wLjAwMDAiLCJQIjoiV2luMzIiLCJBTiI6Ik1haWwiLCJXVCI6Mn0%3D%7C3000%7C%7C%7C&sdata=t3AW%2BWNtVzh%2ByLogyMbb7lRGAa3LmBQKBUq5rmt2pUM%3D&reserved=0) and 
[https://journals.plos.org/plosone/s/file?id=ba62/PLOSOne_formatting_sample_title_authors_affiliations.pdf](https://eur01.safelinks.protection.outlook.com/?url=https%3A%2F%2Fjournals.plos.org%2Fplosone%2Fs%2Ffile%3Fid%3Dba62%2FPLOSOne_formatting_sample_title_authors_affiliations.pdf&data=05%7C02%7Cosm25%40bath.ac.uk%7Cfb6909f2e9014de0667408dc1cbff08c%7C377e3d224ea1422db0ad8fcc89406b9e%7C0%7C0%7C638416858198392545%7CUnknown%7CTWFpbGZsb3d8eyJWIjoiMC4wLjAwMDAiLCJQIjoiV2luMzIiLCJBTiI6Ik1haWwiLCJXVCI6Mn0%3D%7C3000%7C%7C%7C&sdata=Gg%2Bjnc599Xj4CGfDIz1BQqyggPLrw2xYlrHV0XInVMc%3D&reserved=0)

Author response: We have read PLOS ONE’s style requirements and followed these guidelines for the manuscript and supporting information. The figures have been uploaded to the PACE digital diagnostic tool.

2. Thank you for stating the following in the Acknowledgments Section of your manuscript: "ELSA is funded by the National Institute on Aging (R01AG017644), and by UK Government Departments coordinated by the National Institute for Health and Care Research(NIHR)."
We note that you have provided funding information that is not currently declared in your Funding Statement. However, funding information should not appear in the Acknowledgments section or other areas of your manuscript. We will only publish funding information present in the Funding Statement section of the online submission form. 
Please remove any funding-related text from the manuscript and let us know how you would like to update your Funding Statement. Currently, your Funding Statement reads as follows: "This work was supported by an Economic and Social Research Council ([https://www.ukri.org/councils/esrc/](https://eur01.safelinks.protection.outlook.com/?url=https%3A%2F%2Fwww.ukri.org%2Fcouncils%2Fesrc%2F&data=05%7C02%7Cosm25%40bath.ac.uk%7Cfb6909f2e9014de0667408dc1cbff08c%7C377e3d224ea1422db0ad8fcc89406b9e%7C0%7C0%7C638416858198397055%7CUnknown%7CTWFpbGZsb3d8eyJWIjoiMC4wLjAwMDAiLCJQIjoiV2luMzIiLCJBTiI6Ik1haWwiLCJXVCI6Mn0%3D%7C3000%7C%7C%7C&sdata=FUu%2BmEObvXrlS4bUdff8quhWE9aNj8145puvuiphWt8%3D&reserved=0)) funded studentship through the South West Doctoral Training Partnership [grant number ES/P000630/1 to O.S.M.]. The funders had no role in study design, data collection and analysis, decision to publish, or preparation of the manuscript."

Please include your amended statements within your cover letter; we will change the online submission form on your behalf.

Author response: Please find the revised funding statement below:

ELSA is funded by the National Institute on Aging (R01AG017644), and by UK Government Departments coordinated by the National Institute for Health and Care Research (NIHR).

The work for the present study was supported by an Economic and Social Research Council (https://www.ukri.org/councils/esrc/) funded studentship through the South West Doctoral Training Partnership [grant number ES/P000630/1 to O.S.M.]. The funders had no role in study design, data collection and analysis, decision to publish, or preparation of the manuscript.

3. Please review your reference list to ensure that it is complete and correct. If you have cited papers that have been retracted, please include the rationale for doing so in the manuscript text, or remove these references and replace them with relevant current references. Any changes to the reference list should be mentioned in the rebuttal letter that accompanies your revised manuscript. If you need to cite a retracted article, indicate the article’s retracted status in the References list and also include a citation and full reference for the retraction notice.

Author response: We have reviewed our reference list to ensure that it is complete and correct. Please note that, in line with reviewer suggestions, there have been some changes to the reference list from the original submission, including the addition of some new references, and subsequent revisions to the numeric order of the references (these have been indicated with Tracked Changes in the marked-up copy of the manuscript).

Point-by-point response to Reviewer 1’s comments:

Reviewer #1: Thank you having the opportunity to read the intriguing manuscript. The topic of the research (social inequalities in internet use among older individuals) is highly relevant and research on that field is still evolving. The study is based on high quality data and applies a superior method. I very much appreciate the longitudinal perspective as well as the SROBE-statement. However, the manuscript could be improved.

Author response a: We thank the reviewer for these encouraging comments.

Before I come to my suggestions for revision, I would like to point out that I am not an expert in the method used (LCA). However, I have tried to familiarize myself with the method and review the manuscript and the analyses to the best of my ability and conscience.

- The literature section needs to be revised. Please summarize the literature relevant for your argumentation and, based on that, elaborate your study design and research goals

Author response b: Thank you for the positive appraisal of our work. We have elaborated on the research gap and summarised the literature more thoroughly in the introduction section. We have included some additional references and taken care that each step of our argument is supported. Please see Author response 1, 7, and 9 in the table below for further details.

- Some references do not seem to be well chosen.

Author response c: Thank you for raising this – we have made changes in line with your recommendations. Please see Author response 5-7, 9, and 21 in the table below.

- What role plays the COVID-19 pandemic in your study (accelerator for digitalization processes or impacts on health, impacts on economy,…)? Why are you interested in socio-economic outcomes of different purposes of internet use? And why do you use three distinct variables to depict the socio-economic situation? To what extend are they distinct from each other? Please strengthen your “story”/argumentation to underscore the relevance of your paper

Author response d: The COVID-19 pandemic provides a marker in time for this study. We are interested in the “influence of the COVID-19 pandemic on widening or reducing complex inequalities across a broader range of online activities”. We have elaborated on the role of COVID-19 in the introduction (please see Author response 7 in the table below). We use three different measures of socio-economic status to reflect that it is a complicated, multi-faceted construct. There is a rich literature on the complex interplay between education, occupational class and wealth, which is beyond the scope of this work. We have elaborated on our decision to use three separate indicators in the introduction section; please see Author response 9 in the table below.

- Why are the analyses conducted separated for men and women? Please provide a sound argumentation. Are you identifying similar sub-groups if you would have conducted analyses separated for men and women? The information that LCA is conducted separately for men and women pops up abruptly for the reader.

Author response e: As stated in our abstract and methods section, this was based on observed differences according to biological sex “Preliminary analyses explored differences between men and women across the eight types of internet activities, using Pearson’s chi-squared (χ2) test (S3 and S4 Tables). Given the significant differences found at both timepoints (across six of the eight activities), all subsequent statistical analyses were performed separately for male and female participants.” The decision to present models stratified by sex was a result rather than a pre-determined plan. Please see Author response 17 in the table below.

- Why is it important to increase the internet use for different purposes, as we know nothing about the reasons for comparatively lower use

Author response f: We have elaborated on this in the introduction (please see Author response 7 and 9 in the table below). The assertation that internet use for different purposes is important is supported by references 9-11. Arguably, investigating the diversity of internet activities (i.e., different user typologies) offers a more nuanced understanding of online participation among older adults, ensuring that we move beyond simply evaluating inequalities in access, and towards use and benefit.

- The analyses appear to have been carried out thoroughly and in accordance with existing standards

Author response g: Thank you for the positive appraisal of the analyses.

(Please find more detailled comments in the document attached.)

Author response h: Please find our answers to the more detailed comments from the attached document in Table 1 below. Please note that all line numbers match up to the line numbers in the marked-up main document file with tracked changes.

**Table 1.** Point-by-point response to Reviewer 1’s comments (from the attached document)

| **line** | **comment** |
| --- | --- |
| 26 & 50 | Abstract & Introduction   - The literature review is rather thin. I’m aware that this paper uses an explorative approach, however, to elaborate the value of your study you need to provide a sufficient overview over existing literature - The research gap and the studies contribution to the field could be strengthened |
| Author response 1 | We have added detail to the introduction section, to improve the framing of the research gap and this study’s contribution to the field. Please see Author response 7 and 9 for more information on the changes we have made in response to your feedback. |
| abstract | A high degree of stability in latent class membership was observed over **time.However**, men experienced a stark decrease in online health information-**seeking.Our** results highlight that inequality regarding the range of functional and social opportunities provided by the internet prevailed during the pandemic.   - Please check if you forgot some spacings |
| Author response 2 | Thank you for highlighting these; we have checked and the spacing is correct in the main document file submitted, as well as the PDF generated by the PLOS ONE submission system. |
| 27 | “Understanding digital exclusion in vulnerable populations, such as older adults, during the COVID-19 pandemic could help tailor responses to future outbreaks.”   - Please pay attention avoiding ageist language (older adults != vulnerable). |
| Author response 3 | Thank you for bringing this to our attention. This (“vulnerable populations”) has now been removed in line 38 of the abstract. |
| 28 | (also abstract and keywords)   - Are you really doing a cohort study? (no control group) |
| Author response 4 | The authors did not conduct a cohort study, but carried out a secondary analysis of ELSA, which as a prospective cohort study, that follows a group of research participants over time, does not have a control group. We have ensured that the methods for our study have been clearly described. |
| 60 | Older adults are stereotypically described as a technology-resistant, homogeneous group of individuals [4,10].   - Please check if you cited the papers correctly. Do the papers provide empirical evidence for older adults are stereotypically described as a technology-resistant homogeneous group? I don’t think so. It seems like the studies cited use variations of this statement in their introductions, to build their study on a general discussion.   If you want to refer to this general statement, you do not need a text reference.  If you want to use a reference either, it needs to be something like a discourse analysis that finds this prescription of older adults in media / public discussion / … |
| Author response 5 | Thank you for raising this point. We have removed the in-text citations from line 73 and amended the reference list (and order of the numbered references where necessary). |
| 76 | Although a sole emphasis on digital means of social interaction could perpetuate ageism [15,16], the vital role of, and the projected increased reliance on, the internet during and beyond the COVID-19 pandemic may disadvantage older citizens who are offline or not conversant in contemporary technologies [9,23].   - The argument (digital means of social interaction perpetuates ageism) is not clear/intuitively. Please elaborate your argumentation. |
| Author response 6 | We agree that this sentence lacked clarity, so have removed the first part (“Although a sole emphasis on digital means of social interaction could perpetuate ageism”) in line 99-100. |
| 83 | To date, most work has focused on correlates and outcomes of internet access [6], whereas research delving into the specific activities for which the internet is used among older adults is more limited, despite some evidence of differential uptake of internet behaviours relative to younger age groups [25].   - Please investigate literature again. Thera are plenty of studies investigating different purposes of internet use… |
| Author response 7 | Thank you for this helpful recommendation. We have investigated the literature more thoroughly and added detail to this section of the introduction (line 105-115 and 144-153). We have also reframed the beginning of the paragraph and removed the statement that “research delving into the specific activities for which the internet is used among older adults is more limited” from line 106-107 to prevent this from being misleading. |
| 95 | which accelerated the digitalisation of many services.   - Consider removing “of many services”. Then your statement is more openly formulated. |
| Author response 8 | Thank you for this suggestion. We have removed “of many services” from line 144. |
| 102 | A recent study explored differences in internet usage patterns among adults (aged 18-70+) in 104 the Netherlands during the COVID-19 pandemic [31]. Education, but not economic resources, was positively associated with information and communication uses [31]. Nonetheless, previous work has shown that income is particularly important in explaining occupational and consumptive uses [27,32], which were not assessed. As such, the influence of the COVID-19 pandemic on widening or reducing inequalities across a broader range of online activities remains unknown.   - Please revise your argumentation and read the literature more carefully (which other control variables are used in that study? Do you think income is similar to economic resources / other “socio-economic variables…?) - Please make clear what you mean by “socio-economic” aspects in your study? What are you looking at? Explain why you think different variables are related to the same concept and what makes them distinct to each other - Please check if the studies you cite provide evidence for the directionality of associations and check if COVID-19 is evidenced as cause of an effect |
| Author response 9 | Thank you for these suggestions.   - We have now clarified that “economic resources” was defined as participants’ annual family income in the last 12 months in line 161-162. - We have clarified what we mean by socio-economic status and explained why it is important to look at multiple indicators in line 165-194. - We added that the study was looking at COVID-19-related internet uses in line 160 (to avoid any confusion of COVID-19 as the cause). - We have amended the section to clarify the directionality of associations for other studies exploring income as a correlate of internet use in line 163-165. |
| 119 | **Study design and participants**   - Both surveys underlying the analyses of this manuscript belonging to ELSA. However, even if the surveys are very similar and similar or identical questions are asked of the same individuals at different times (2018/19 and June/July 2020), there are still differences that can influence the results of the study. In addition to differences in the formulation of the questions and the response categories, the change in the survey mode (from CAPI to Online Survey or CATI) certainly poses the greatest challenge for the present study (that is interested in internet-use).   Perhaps there is already a publication that examines the mode effects of the ELSA surveys (or similar surveys) and can be referred to here? Moreover, the differences between the surveys and possible effects on the study could be discussed in more detail (in the study design section or in limitations section). |
| Author response 10 | We agree that the mode of data collection, as well as differences in the formulation of the questions and response categories could have consequences for the study results and have outlined these in the limitations section of the discussion in line 613-618. Seeing as the participants contributing to the present study are all internet users (i.e., those who reported “never” using the internet were excluded, as the response options on different purposes of internet use were not applicable to them), the difference in survey mode may not be as relevant as if we were exploring the frequency of internet use (and therefore including participants who reported “never” using the internet in analyses). Participants did also have the option to complete the survey via telephone interview, which is described on line 223. |
| 123 | This sentence may be deleted: “The authors did not have access to personal data.”   - Information could be placed in the “Additional Information”-section |
| Author response 11 | We have included this statement as it is a journal requirement by PLOS ONE and was requested when the article went through the initial quality checks (in line with the Human Participants Research Checklist). |
| 131 | Select**ed ?** |
| Author response 12 | Thank you for highlighting this; we have changed this to “participants selected from” in line 221 to improve the clarity of the sentence. |
| 139 | If a definition of the "core members" of the ELSA survey is teased (because relevant for your article), please include it and not just make a reference to the documentation. |
| Author response 13 | Thank you for the suggestion. We have now added the eligibility criteria for ELSA core members in line 228-231. |
| 147 | **Measures** |
| 150 | What time reference is used in the question on average frequency of internet or email usage? Please add. |
| Author response 14 | We have checked and there was no specific time reference. The question in quotation marks on line 254-255 (‘On average, how often do you use the internet or email?’) is exactly as it features in the ELSA questionnaire. In the COVID-19 sub-study, participants were asked (‘Since the coronavirus outbreak, on average, how often did you use the internet or email?’), as indicated in line 255-257. |
| 154 | For a better overview, the comparison of the response categories of the internet-use-questions of ELSA 2018/19 and ELSA COVID 2020 could be displayed reader-friendly in a table (like you did in table 1). |
| Author response 15 | As frequency of internet use is not a main variable of interest (it is mainly included within the manuscript, as we mention at the end of that section that “Respondents who reported never using the internet (~19%) were excluded from analyses, as the response options representing different purposes of internet use were not applicable to them”), we don’t think it is necessary to include a table to display the response options within the main text. However, to improve the comparison of the categories and response options between baseline and follow-up, we have added a table in the Supplementary Material (new S1 Table). |
| 158 | From lines 153 to 155 and table S1 it becomes clear, that answer categories are similar, but differ between the surveys regarding the recorded units of frequency, the number of categories, wording and the examples given.  For example, …  … your generated category “High” comprises individuals that responded “more than one a day” in one survey and “every day, or almost every day”  …your generated category “internet transactions” that comprises the answer categories “finances” and “public services” in the ELSA 2018/19 survey, but only “finances” in the ELSA COVID-19 survey.  Please point out these differences and possible consequences for the results of this study in the text. |
| Author response 16 | We have clarified the response options to the frequency and purpose of internet use questions in S1 Table (please see Author response 15) and S2 Table. In addition, we have commented on the possible consequences of these differences for the results of the study in the limitations section of the discussion in line 611-618. |
| 181 | Preliminary analyses explored differences between men and women across the eight types of internet activities, using Pearson’s chi-squared (𝜒2) test (S2 and S3 Tables).   - Please be precise: not all types of internet activities |
| Author response 17 | Thank you for the suggestion. We have added that statistically significant differences were found across “*six* of the eight activities” at baseline and follow-up in line 295. |
| 236 | Please check and update your link. It doesn’t seem to work properly:  <https://github.com/OliviaMalkowski/Inequalities-Internet-COVID-19.git> |
| Author response 18 | We had kept the link private on purpose and were planning on making it public upon publication of the work (in line with PLOS guidelines for sharing code). Upon your request, we have now made the link public. |
| 237 | Results   - You could list the characteristics of the identified classes in a table. This would make it easier for the reader to grasp the classes along with their |
| Author response 19 | We appreciate the suggestion. However, to prevent repetition, we would prefer to keep the figures in the main text (rather than describe the characteristics in a table), as this is how clusters identified in LCA are typically presented. We would also prefer to keep the description of the figures in the main text (rather than putting this information in a table), as otherwise there will be tables and figures in immediate succession of one another without any link to the text. |
|  | Discussion   - Please elaborate more on why the identification of sub-groups of internet users is relevant in the context of COVID-19 - The finding that health information seeking has decreased among in the COVID-19 wave in comparison to the pre-pandemic level is surprising and interesting. You discuss some plausible explanations. Another explanation could be the overburdening of the healthcare system and postponement of operations and treatments or the absence of medical examinations. Individuals may seek health information on the internet before undergoing surgery or treatment or after receiving diagnoses. - Please consider that not all older individuals may wish to increase their use of the internet (for various purposes). Differences in usage frequency do not necessarily stem from external barriers. To ensure the societal inclusion of all groups of older people, offerings and services should always be available in both digital and analog formats. |
| Author response 20 | Thank you for these suggestions.   - We have added some more plausible explanations for the decline in health information seeking in line 570-584 and have simultaneously elaborated on the relevance of the study in the context of COVID-19. - Furthermore, we have clarified that services and support should be accessible in different formats for older adults who may not wish to increase their internet use (or use the internet at all) in line 648-652. |
|  | Literature |
| 551 | Please check this reference. It’s a preprint. Results are not presented. Please consider using another reference.  Kebede AS, Ozolins LL, Holst H, Galvin K. The digital engagement of older people: systematic scoping review protocol. JMIR Res Protoc. 2021 Jul 5;10(7):e25616. |
| Author response 21 | Thank you for the suggestion. We have replaced this with a more appropriate reference (please see new reference 3, line 750). |
|  | Appendix |
| S1 Appendix | The table is not intuitively to understand, as some information is missing for the reader:   - Please provide a column header for col. 1. - Please add a note that the text written in bold letters in the “recommendation”-column are **added by you** and are referring to the section in the manuscript that covers each respective recommendation of the STROBE guidelines. OR: add an additional column containing references to your text (Item No.-column could be dropped, if there are formatting issues) - Please provide the source of the STROBE table as a note below the table. - Please provide an explanation of what the asterisk indicates. - Please provide a brief description of the STROBE guidelines/statement as a note below the table or in the text. |
| Author response 22 | Thank you for these suggestions.   - The checklist/table was downloaded straight from the official STROBE website (<https://www.strobe-statement.org/checklists/>). Seeing as we are only editing the page numbers/manuscript sections (i.e., the information in bold), we will not change the formatting of the column headers. - We have added a note beneath S1 Appendix to indicate that the text in bold refers to the sections of the manuscript that cover each of the STROBE recommendations. - We have added the source of the STROBE checklist as a note below the checklist. - We have included an explanation of what the asterisk indicates (this statement comes directly from the STROBE publication) as a note below the checklist. - We have added a brief description of the STROBE guidelines in the main text in line 250-251.   Amendments to the checklist notes are visible in the “Supporting Information” section on page 33 (line 972 onwards). |
| Table 1 | Please add information on the precise formulation of the questions in both surveys (in the “notes” below the table).  Please harmonize the wording (“variable” in the title; “item” in the column headers; …“response options” in the text). |
| Author response 23 | We have added the precise formulation of the questions in both surveys in the notes below S1 Table and S2 Table.  Thank you for pointing out this discrepancy. We have now changed any mention of “variable” or “item” to “response options” in S1 Table and S2 Table. We have also ensured that this is consistent in the main text, with changes made in line 266, 270, and 277. The amendments to the table titles and notes are visible in the “Supporting Information” section on page 33 (line 972 onwards). |
| S2 & S3 | In addition, please provide information on your data source in the table notes. |
| Author response 24 | The data source (i.e., ELSA) is described in the main manuscript, and for consistency, is only included in the notes beneath tables if the abbreviation has been used in the table(s) or table title(s). |
| Fig 1 | The resolution could be improved to make the figure looking more professional. |
| Author response 25 | Thank you for raising this. The compiled submission PDF includes low-resolution preview images of the figures only. The figures can be downloaded from the generated PDF in high-resolution. The original files uploaded meet the resolution requirements (and will be of much higher quality in the eventual manuscript relative to the compiled submission PDF). |

Point-by-point response to Reviewer 2’s comments:

Reviewer #2: The article addresses an interesting topic. I can make a relevant contribution to PLOS ONE and the digital inequalities area. I agree that research looking at how different activities on the internet are related to different characteristics and factors in relevant to understand digital inequalities of older people. I miss the inclusion of more factors in the final analysis. For instance, we know that among the older population, age matter. The manuscript focuses on discussing self-reported biological sex, and the observed difference may be a consequence of other factors too (i.e. age, physical and mental health, ethnicity, etc.).
I miss some additional references to studies that have also looked at activities older people do online.

Fernández-Ardèvol, M., Rosales, A. and Cortès, F.M., 2023. Set in stone? Mobile practices evolution in later life. Media and Communication, 11(3), pp.40-52.

Beneito-Montagut, R., Rosales, A. and Fernández-Ardèvol, M., 2022. Emerging digital inequalities: A comparative study of older adults’ smartphone use. Social Media+ Society, 8(4), p.20563051221138756

Author response: We thank the reviewer for their comments. We did indeed control for age in our analyses. This is specified in line 290-291, 324, and 345. Furthermore, the analyses are stratified by biological sex. This is stated in line 293-296. We did not control for more covariates, as conditional LCA and LTA methods are computationally intensive. The ELSA sample is predominantly White, and we have commented on this in line 618-619. However, we acknowledge the potential influence of other covariates and residual confounding. We have added a sentence in the limitations section of the discussion to this effect in line 620-622. Thank you for the references; these have been integrated at various points in the introduction (line 113-115 and 144-153).
