# Supplementary material for: Socio-economic inequalities in the breadth of internet use before and during the COVID-19 pandemic among older adults in England
Source: PLoS One. 2024 May 9;19(5):e0303061. doi: 10.1371/journal.pone.0303061 (PMC11081243; doi:10.1371/journal.pone.0303061)
Supplement: Attachment — Submitted filename: Response to Reviewers.docx [file pone.0303061.s015.docx]

Dear Editor,

Thank you for the opportunity to revise the manuscript titled “Socio-economic inequalities in the breadth of internet use before and during the COVID-19 pandemic among older adults in England”. We appreciate the reviewer’s follow-up suggestions to improve the quality of the manuscript. To address feedback on the discussion section, we have reiterated that other explanations for differences in the latent classes over time may exist, beyond the COVID-19 pandemic and the switch in the mode of data collection; we have also used suggestive language throughout, so as not to infer causality. Furthermore, we have taken on board the reviewer’s feedback regarding the use of brackets, and have deleted unnecessary text and/or removed brackets where appropriate to streamline the manuscript.

Journal Requirements:

1. Please review your reference list to ensure that it is complete and correct. If you have cited papers that have been retracted, please include the rationale for doing so in the manuscript text, or remove these references and replace them with relevant current references. Any changes to the reference list should be mentioned in the rebuttal letter that accompanies your revised manuscript. If you need to cite a retracted article, indicate the article’s retracted status in the References list and also include a citation and full reference for the retraction notice.

Author response: We have reviewed our reference list to ensure that it is complete and correct. We have also checked that none of the cited papers have been retracted. No changes have been made to the reference list during this round of revisions.

Point-by-point response to Reviewer 1’s comments:

Reviewer #1: I am pleased that the feedback has been carefully reviewed and the manuscript revised. The manuscript has gained in strength and clarity.

Author response a: We thank the reviewer for these encouraging comments.

However, I would like to make two points:

1. Please consider that the differences in latent classes may not have been evoked only by the COVID-19 pandemic or a mode effect. The study dates make it possible to speak of "pre-pandemic" and "during the COVID-19 pandemic", but the COVID pandemic can only be discussed as an explanation. (lines 516-518)

Author response b: Thank you for raising this. We have ensured that the discussion section reflects that the COVID-19 pandemic and the mode of data collection are not the only plausible explanations for differences in the latent classes over time. Correspondingly, we have made changes to lines 495-497 and 546-548.

2. The authors very often use brackets to add additional information. If the information in the brackets is not essential, it can simply be deleted from the text. If the information is important, it does not have to be in brackets. For me as a reader, it is easier if the authors decide what is really relevant information in the main text and what is actually not so important and dispensable. In this way, the authors could streamline and shorten their text a little.

Author response c: Thank you for this suggestion. Accordingly, we have either deleted the text if the information was not essential or removed the brackets if the information was deemed important. Changes have been made to the following lines: 51, 70-71, 94, 97, 112-114, 122, 124-126, 159, 161, 187-188, 229-230, 235, 245, 362, 373-374, 431, 437, 445, 457-458, 461, 467-468, 543-544, 577, 802-803, 822-823, and 827-828.
